# An Exploratory Assessment of Early Feeding Modality and Associated Stomatognathic, Occlusal, and Language Outcomes in Preterm Children: An Observational Cross-Sectional Study

**DOI:** 10.3390/diagnostics16172727

**Published:** 2026-08-26

**Authors:** Ștefan Lucian Burlea, Laura Elisabeta Checheriță, Ovidiu Stamatin, Loredana Golovcenco, Vlad Ștefan Proca, Maria Antonela Beldiman, Gabriel Goian, Tudor Hamburda, Violina Budu, Bogdan Petru Bulancea, Anamaria Ciubară, Crînguța Mariana Paraschiv, Liana Aminov, Oana-Irina Gavril, Ana Elena Sîrghe

**Affiliations:** 1 3rd Dental Medicine Department, Faculty of Dental Medicine, “Grigore T. Popa” University of Medicine and Pharmacy, 700115 Iași, Romaniamariaantonelabeldiman@umfiasi.ro (M.A.B.); bogdanbulancea@umfiasi.ro (B.P.B.); 2 2nd Dental Medicine Department, Faculty of Dental Medicine, “Grigore T. Popa” University of Medicine and Pharmacy, 700115 Iași, Romaniatudorhamburda@umfiasi.ro (T.H.); violinabudu@umfiasi.ro (V.B.); lianaaminov@umfiasi.ro (L.A.); 3 1st Dental Medicine Department, Faculty of Dental Medicine, “Grigore T. Popa” University of Medicine and Pharmacy, 700115 Iași, Romaniaana.sirghe@umfiasi.ro (A.E.S.); 4 PH DRD Discipline of Phixed Prosthesis, “Grigore T. Popa” University of Medicine and Pharmacy, 700115 Iași, Romania; gabigoian@yahoo.com; 5 Psychiatry Department, Faculty of General Medicine, “Dunărea de Jos” University of Medicine and Pharmacy, 800216 Galați, Romania; 6 Department of Medical Specialties (I), Faculty of Medicine, “Grigore T. Popa” University of Medicine and Pharmacy, 700115 Iași, Romania

**Keywords:** stomatognathic dysfunction, cross-sectional study, early diagnosis, prophylaxis, breastfeeding, occlusal development, language acquisition, preterm infants, non-nutritive sucking, parenteral nutrition, digestive and stomatognathic system

## Abstract

**Background/Objectives:** Malocclusion and delayed expressive language are generally managed as separate clinical problems, although both may be influenced by early feeding experiences and stomatognathic development. This study investigated whether neonatal feeding modality was associated with occlusal morphology and expressive language development in preterm children, and explored the relationship between these outcomes. **Methods:** Forty preterm children (gestational age < 37 weeks) stratified: breastfeeding (Group A, n = 14), tube feeding with structured non-nutritive sucking (NNS) (Group B, n = 13), and total parenteral nutrition without oral stimulation (Group C, n = 13). Seven of 47 children assessed were excluded according to predefined eligibility criteria. Occlusal morphology was independently evaluated by two blinded paediatric dentists (Cohen’s κ = 0.91). Expressive language was evaluated using the MacArthur–Bates Communicative Development Inventories and a structured phonological checklist. Group differences were analysed using Kruskal–Wallis and Dunn–Bonferroni tests; associations were examined using Spearman ‘s rank correlation and multivariable ordinary least squares regression adjusted for sex, area of residence, and socioeconomic status. **Results:** Significant differences were observed among the three feeding groups in occlusion score, language score, and age at first words (all *p* < 0.001; ε^2^ = 0.81–0.90). Occlusion and language scores were strongly associated (Spearman’s ρ = 0.986, *p* < 0.001), and this association remained essentially unchanged after adjustment for demographic covariates (β = 2.10, 95% CI 2.01–2.19, *p* < 0.001). Occlusal morphology subtypes also differed significantly across groups (χ^2^(6) = 19.1, *p* = 0.004). **Conclusions:** Neonatal feeding modality was associated with both occlusal morphology and expressive language outcomes in this cohort of preterm children. Breastfeeding was associated with the most favorable outcomes, structured NNS with intermediate outcomes, and TPN with the least favorable outcomes. Given the observational design, modest sample size, and potential for residual confounding, findings should be considered exploratory and require confirmation in larger prospective multicenter studies.

## 1. Introduction

Preterm birth—defined as delivery before 37 completed weeks of gestation—affects approximately one in ten live births worldwide and is associated with increased risks across multiple developmental domains, including feeding difficulties, craniofacial structural abnormalities, and delayed communicative development [1]. Although these outcomes are conventionally assessed and managed by different clinical specialists—neonatologists, pediatric dentists, and speech-language pathologists—emerging evidence suggests they may share biological pathways related to early feeding experience. This question has potential clinical relevance because if a single modifiable neonatal exposure is associated with both structural and linguistic outcomes, this could support consideration of integrated screening and preventive strategies that are currently absent from most neonatal follow-up protocols.

To understand why feeding modality might influence both jaw structure and early language development, it’s useful to be seen stomatognathic system as an integrated functional unit encompassing the structures, muscles, and neural circuits On the mechanical side, breastfeeding promotes jaw and facial growth through coordinated sucking movements, while on the biochemical and nutritional side, breast milk provides essential nutrients and growth factors that support brain and craniofacial development. These benefits may be reduced with nasogastric feeding (where milk is delivered directly into the stomach via a tube) or total parenteral nutrition (TPN), in which nutrients are delivered directly into the bloodstream, thereby bypassing oral feeding [2,3,4,5,6,7]. These mechanistic pathways are described here as background context based from prior studies [1,2,3,4,5,6,7]; they were not directly assesd in the present cohort and should therefore not be interpreted as findings of this study.

Previous studies have reported associations between early feeding practices and occlusal development, although some aspects of this relationship remain under investigation. The most consistently replicated finding—documented in systematic reviews by Boronat-Catalá et al. (2017) [8] and Peres et al. (2020) [9], and in longitudinal cohorts by Warren et al. (2005) [10] and Moimaz et al. (2021) [11]—suggest that longer or exclusive breastfeeding beyond four to six months reduces the prevalence of posterior crossbite and maxillary hypoplasia in primary dentition. The mechanistic explanation draws on the functional matrix hypothesis first proposed by Moss (1997) [12]: skeletal form is driven primarily by the functional demands of surrounding soft tissues, so that reduced jaw-muscle loading—as occurs with bottle, tube, or parenteral feeding—attenuates sutural growth and arch widening. Quantitative support for this framework has come from Kiliaridis (1995) [13], who showed across animal and human studies that masticatory muscle hypofunction leads to measurably narrower maxillary arches, and more recently from Liang et al. (2024) [14], whose finite-element modelling of infant crania demonstrated that masticatory forces triple in magnitude between birth and 48 months, progressively concentrating mechanical strain on the facial skeleton in precisely the pattern that breastfeeding drives from the earliest weeks of life. An ongoing question within this literature is whether non-nutritive sucking habits (pacifier or digit) amplify or merely modify the protective effect of breastfeeding on occlusal development. Leite-Cavalcanti et al. [15] and Chen et al. (2024) [16] have reported that such habits attenuate the breastfeeding benefit, whereas other investigators maintain that the duration and exclusivity of breastfeeding remain the dominant predictors regardless of pacifier use [8,11].

Early nutrition functionally links the digestive and stomatognathic systems (SS), supporting physiological homeostasis, craniofacial growth, oral function, and neurodevelopment. Early feeding experiences may therefore influence multiple aspects of postnatal development [1,3,12].

A growing body of epidemiological research has also linked early feeding practices to language development, although the underlying mechanisms remain actively debated.whereas the landmark global synthesis by Victora et al. (2016) [1] situated these linguistic advantages within the broader pattern of breastfeeding’s neurodevelopmental benefits. Several biological pathways have been proposed, although their relative contributions remain uncertain. The first is nutritional: DHA and AA accumulate preferentially in cortical grey matter and synaptic membranes during the last trimester and first two postnatal years [17], and randomised trials have established that neonatal DHA supplementation improves intelligence test scores at five years in preterm children [18] and neurodevelopmental scores at 18–22 months corrected age [19]. The second is neuromotor: neuroimaging studies have shown that early oral-motor activity recruits cortical regions overlapping with Broca’s area and adjacent premotor cortex [20], raising the possibility that the thousands of coordinated sucking cycles performed during breastfeeding constitute an early form of training for the same neural networks later engaged in phonological planning [21]. These two pathways are not mutually exclusive, and their respective contributions remain unquantified—an acknowledged limitation of the current literature [3,17].

Despite the parallel growth of these two research streams, a critical gap persists: the two literatures have developed largely in isolation, and no study has directly quantified how strongly occlusal morphology and expressive language co-vary within the same controlled cohort. This gap is not inconsequential, as two competing interpretations remain open. One interpretation is that feeding modality influences occlusal and language outcomes independently—both downstream of the same upstream cause—so that any correlation between the two outcomes would be modest and largely explained by demographic confounders. An alternative hypothesis, consistent with the neurofunctional model proposed by Smith and Goffman in Mason et al. (2011) [22], suggests that occlusal morphology may influence articulatory performance and therefore could be associated with early language development. However, this relationship has not yet been directly established in preterm cohorts. A second unresolved question concerns non-nutritive sucking habits (NNS) specifically: randomised evidence from Alidad et al. (2021) [23] and a meta-analytic synthesis by Zhao et al. (2024) [24] confirm that structured NNS during tube feeding accelerates the transition to independent oral feeding in preterm neonates, but whether NNS also attenuates the craniofacial and linguistic consequences of the absence of breastfeeding has not been tested in a cohort encompassing all three feeding routes.

Preterm infants provide a particularly informative population in which to address these questions. They have an elevated baseline risk of both malocclusion and language delay [25,26,27,28,29] with naturally occurring, clinically driven variability in feeding modality—from fully breastfed to exclusively parenterally fed—that cannot be ethically assigned by randomisation. We therefore examined a consecutive observational cohort of 40 preterm children stratified according to neonatal feeding route—breastfeeding (Group A, n = 14), enteral feeding with structured NNS (Group B, n = 13), or TPN without oral stimulation (Group C, n = 13)—to pursue three pre-specified objectives: (I) compare occlusal morphology, expressive language development, and age at first words among preterm children exposed to different neonatal feeding modalities; (II) evaluate the association between occlusal development and language outcomes within the study cohort; and (III) explore whether this association remained after adjustment for selected demographic variables (sex, area of residence, and socioeconomic status). We hypothesised that more favourable early feeding conditions would be associated with better occlusal and language outcomes, while recognizing that the observational design does not permit causal inference [6,7]. These findings may contribute to the development of integrated stomatognathic screening and preventive strategies in neonatal follow-up programmes.

## 2. Materials and Methods

This manuscript is reported in accordance with the Strengthening the Reporting of Observational Studies in Epidemiology (STROBE) statement for cross-sectional studies (Supplementary File S1)[30].

### 2.1. Study Design and Setting

We conducted a single-centre, observational, cross-sectional study. Children were recruited consecutively from two affiliated pediatric developmental follow-up programmes at the ‘Grigore T. Popa’ University of Medicine and Pharmacy, Iași, Romania between 17 April 2024 and 31 March 2026. The study was approved by the Ethics Committee of the ‘Grigore T. Popa’ University of Medicine and Pharmacy, Iași (protocol No. 172, approved 17 April 2022) and conducted in full accordance with the Declaration of Helsinki. Written informed consent was obtained from the legal guardian of each participant before any data were collected (Supplementary File S2).

### 2.2. Participants and Sampling

**Figure 1:**
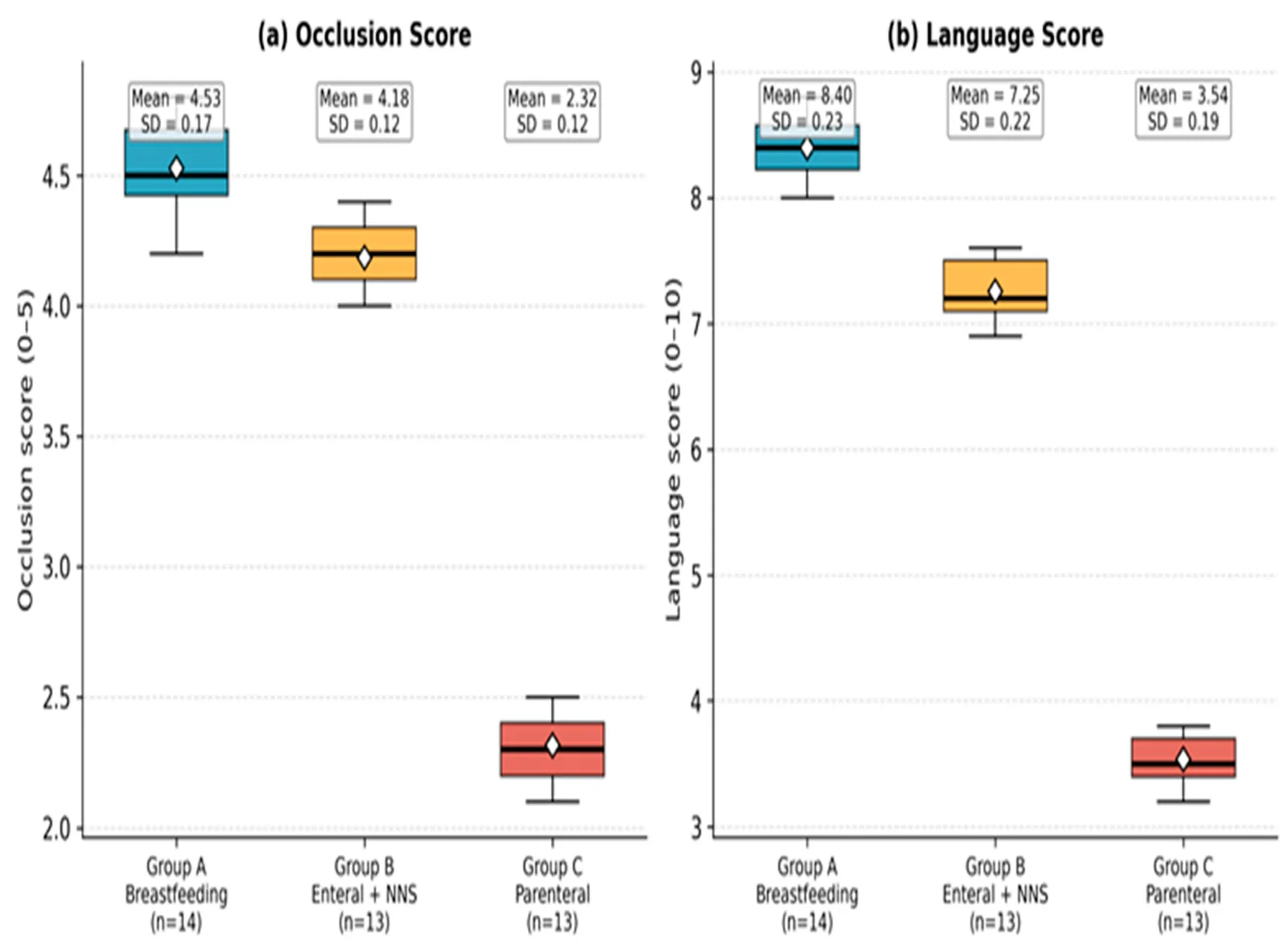
Distribution of occlusion (**a**) and language (**b**) scores according to neonatal feeding modality. Boxes represent the interquartile range (IQR), horizontal lines indicate the median, whiskers represent the observed range, and dots indicate individual participants. Group A = breastfeeding; Group B = enteral feeding with structured non-nutritive sucking; Group C = total parenteral nutrition without structured oral stimulation.

To be eligible, children had to have been born before 37 completed weeks of gestation (gestational age [GA] < 37 weeks), be aged 36–42 months corrected age at assessment, and have complete neonatal feeding documentation covering the first six months of life. We applied three pre-specified exclusion criteria: (i) congenital maxillofacial malformation; (ii) a diagnosed neurological syndrome; and (iii) endotracheal intubation lasting more than three months, as each of these could independently affect occlusal or language outcomes regardless of feeding modality. All eligible children attending the two programmes during the recruitment window were consecutively invited to participate; no probability-based sampling method was applied. Of 47 children assessed for eligibility, 7 were excluded. The participant selection process is presented in the Participant Flow Diagram (Supplementary File S3). The remaining 40 were stratified by their documented neonatal feeding modality (Figure 1):

- Group A—Breastfeeding (n = 14): exclusive breastfeeding ≥4 months.

Group A (n = 14): exclusive breastfeeding for at least the first four months of life, followed by continued breastfeeding with age-appropriate complementary feeding between months 4 and 6, according to WHO recommendations [1].

- Group B—Enteral feeding with structured NNS (n = 13): nasogastric/orogastric tube feeding with a structured NNS programme (≥3 sessions/day).
- Group C—Parenteral nutrition only (n = 13): TPN with no structured oral stimulation.

No child who was invited declined to participate, and no participant was lost to follow-up before assessment; accordingly, all 40 enrolled children contributed to the final analysis.

### 2.3. Variables

The primary exposure was neonatal feeding modality (three-level categorical: Groups A, B, C). The three co-primary outcomes were occlusion score (continuous, 0–5 scale, higher = more favorable morphology), expressive language score (continuous, 0–10 scale), and age at first words (continuous, months). A secondary categorical outcome was descriptive occlusion subtype (normal occlusion, anterior open bite, posterior crossbite, or deep bite). We pre-specified three candidate confounders on biological and epidemiological grounds: sex (binary: male/female), area of residence (binary: urban/rural), and SES (three-level ordinal: low/medium/high). Because of the limited sample size, adjustment was restricted to prespecified demographic variables to reduce model overparameterization. Other clinically relevant variables were considered during interpretation and are discussed as potential sources of residual confounding.

### 2.4. Occlusal Assessment

Intraoral digital scans were acquired using a TRIOS 3Shape intraoral scanner (3Shape A/S, Holmens Kanal 7, 1060 Copenhagen, Denmark). Two calibrated pediatric dentists—each blinded to the child’s feeding group—then independently scored each scan on a five-point morphometric scale: 0 represented severe malocclusion with multiple structural abnormalities, and 5 represented normal occlusion with full molar symmetry, adequate palatal width, and no crossbite. Inter-rater reliability was excellent (Cohen’s κ = 0.91). The few discrepancies observed were limited to one-point differences and were resolved by consensus.

### 2.5. Language Assessment

All language assessments were conducted by a certified speech-language pathologist who was blinded to each child’s feeding group allocation. The evaluation drew on three complementary sources: (I) the MacArthur-Bates CDI, in its locally validated Romanian version, from which a composite score (0–10) was derived covering vocabulary, sentence production, and morphological complexity; (II) a structured phonological checklist administered during a standardised play session, targeting 20 consonantal phoneme contrasts, five sentence-construction tasks, and a 10-item lexical diversity probe; and (III) age at first words, obtained through structured caregiver interview and cross-validated against the child’s existing pediatric developmental records.

### 2.6. Bias

We addressed bias at the design stage through blinding: both dental examiners and the speech-language pathologist were unaware of each child’s feeding group when scoring (κ = 0.91 quantifies the residual information uncertainty in occlusal scoring). Potential confounding by sex, area of residence, and SES was controlled analytically by including all three variables as pre-specified covariates in every multivariable model.

We also acknowledge a related source of selection bias: infants requiring prolonged parenteral nutrition are disproportionately drawn from a clinically more vulnerable population. Consequently, feeding modality may partly reflect underlying illness severity rather than acting as an independent exposure; this potential source of residual confounding is addressed further in the Discussion.

### 2.7. Study Size

Sample size was determined pragmatically by the number of eligible children attending the two programmes during the recruitment period, rather than by a formal a priori power calculation. Although post hoc effect estimates were large, these should be interpreted cautiously, owing to the relatively small sample size.

### 2.8. Quantitative Variables

All three primary outcomes—occlusion score, language score, and age at first words—were analysed as continuous variables throughout; we did not apply any post hoc categorisation or transformation. Feeding modality was modelled as a three-level unordered categorical factor. The three confounders were coded as follows: sex (binary: 0 = female, 1 = male), area of residence (binary: 0 = rural, 1 = urban), and SES (ordinal: 0 = low, 1 = medium, 2 = high).

### 2.9. Statistical Methods

Descriptive and inferential statistical methods were used throughout the study. Continuous variables were summarized using medians and interquartile ranges (IQRs), whereas categorical variables were reported as frequencies and percentages.

Because of the relatively small sample size and the non-normal distribution of several study variables, non-parametric statistical methods were used for group comparisons. Differences among the three feeding groups were evaluated using the Kruskal–Wallis H test, followed by Dunn–Bonferroni post hoc tests for pairwise comparisons when the overall test was statistically significant. In addition to *p*-values, epsilon-squared (ε^2^) was calculated as a measure of effect size for the Kruskal–Wallis test.

The association between occlusal development and expressive language performance was assessed using Spearman’s rank correlation coefficient. To examine this relationship while accounting for potential confounding, a multivariable ordinary least squares (OLS) regression model was fitted, with language score as the dependent variable and occlusion score as the main independent variable. Sex, area of residence, and socioeconomic status (SES) were included as covariates. Regression results are reported as unstandardized regression coefficients (B), 95% confidence intervals (95% CI), and *p*-values. Categorical variables were compared using the chi-square test of independence, and Cramér’s V was calculated as the corresponding measure of effect size. Basic regression diagnostics were performed before interpretation of the fitted model. Residual normality was evaluated using the Shapiro–Wilk test together with visual inspection of normal probability (Q–Q) plots. Homoscedasticity was assessed by visual inspection of residual-versus-fitted plots, whereas multicollinearity and influential observations were examined using variance inflation factors (VIFs) and Cook’s distance, respectively. All statistical tests were two-sided, and statistical significance was defined as *p* < 0.05.

Statistical analyses were performed using IBM SPSS Statistics for Windows, Version 27.0 (IBM Corp., Armonk, NY, USA).

## 3. Results

### 3.1. Participant Flow and Baseline Characteristics

Of the 47 preterm children we assessed for eligibility, seven were excluded: three had a congenital maxillofacial malformation, two had a diagnosed neurological syndrome, and two had required endotracheal intubation for more than three months. The remaining 40 enrolled children all completed the full assessment protocol—none declined, and none was lost to follow-up before the assessment visit. No missing data were identified for any primary or secondary outcome. Because socioeconomic status differed among groups, this variable was included as a covariate in the multivariable regression analysis.

Baseline demographic characteristics are shown in Table 1. Sex distribution (χ^2^(2) = 0.34, *p* = 0.844) and area of residence (χ^2^(2) = 0.07, *p* = 0.964) were comparable across the three groups. SES, however, differed significantly (χ^2^(4) = 11.92, *p* = 0.018, Cramér’s V = 0.39), with Group C containing proportionally more low-SES families than Groups A or B. This imbalance is addressed analytically in Section 3.4 and interpreted as a limitation in Section 4.3. SES was therefore retained as a covariate in all multivariable models.

### 3.2. Occlusal Morphology

Occlusion scores differed substantially across the three feeding groups (Table 2, Figure 1a). Children in the breastfeeding group (Group A) had the highest median occlusion score (4.50, interquartile range [IQR] 4.43–4.67), consistent with well-aligned, symmetric dental arches. The enteral feeding plus structured non-nutritive sucking group (Group B) demonstrated intermediate occlusion scores (median 4.20, IQR 4.10–4.30), whereas the parenteral nutrition group (Group C) had the lowest scores (median 2.30, IQR 2.20–2.40), reflecting a predominance of posterior crossbite and high-arched palate morphology. The Kruskal–Wallis test indicated a highly significant overall difference in occlusion score among the three feeding groups (H(2) = 33.1, *p* < 0.001) with a large effect size (ε^2^ = 0.81). All three pairwise comparisons (A vs. B, A vs. C, B vs. C) remained statistically significant after Dunn–Bonferroni correction, with large between-group differences in medians (Table 3).

### 3.3. Language Development

**Figure 2:**
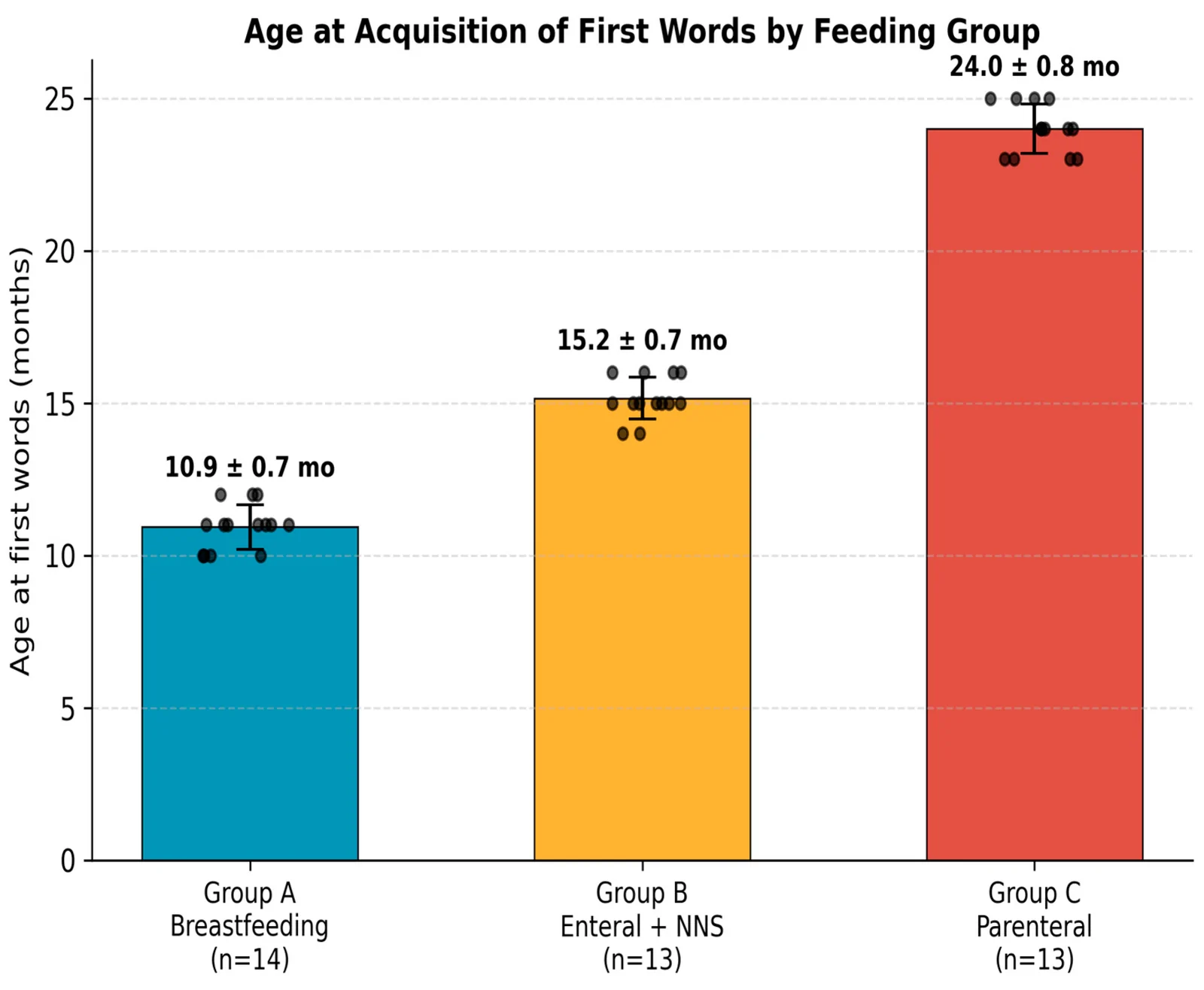
Age at acquisition of first words (months) by feeding group. Bars represent group medians and dots represent individual participants. Group A = breastfeeding; Group B = enteral feeding with structured non-nutritive sucking; Group C = total parenteral nutrition without structured oral stimulation.

Expressive language scores displayed the same ordered pattern as occlusion scores (Table 2, Figure 1b). Expressive language scores displayed the same ordered pattern as occlusion scores (Table 2, Figure 1b). Children in Group A had the highest median language score (8.40, IQR: 8.22–8.57) and reached their first words earliest (median 11 months, IQR: 10–11). Group B showed intermediate language scores (median 7.20, IQR: 7.10–7.50) and acquired first words at a median of 15 months (IQR: 15–16). Children in Group C exhibited the greatest delay, with a median language score of 3.50 (IQR: 3.40–3.70) and first words acquired at a median of 24 months (IQR: 23–25), approximately 13 months later than children in Group A (Figure 2). Both language outcomes differed significantly across the three groups (language score: H(2) = 34.7, *p* < 0.001; age at first words: H(2) = 35.3, *p* < 0.001). The corresponding effect sizes were large (language score: ε^2^ = 0.88; age at first words: ε^2^ = 0.90). Dunn–Bonferroni post hoc comparisons showed significant pairwise differences for both outcomes (Table 3).

These differences were confirmed statistically: the Kruskal–Wallis test for language score yielded H(2) = 34.7 (*p* < 0.001) and the test for age at first words yielded H(2) = 35.3 (*p* < 0.001), with all Dunn–Bonferroni pairwise contrasts reaching significance (*p* < 0.001; Table 3). The median age at first words was 11 months (IQR 10–11) in Group A, 15 months (IQR 15–16) in Group B, and 24 months (IQR 23–25) in Group C, corresponding to a 13-month gap between Groups A and C (Figure 2).

### 3.4. Occlusion–Language Association

**Figure 3:**
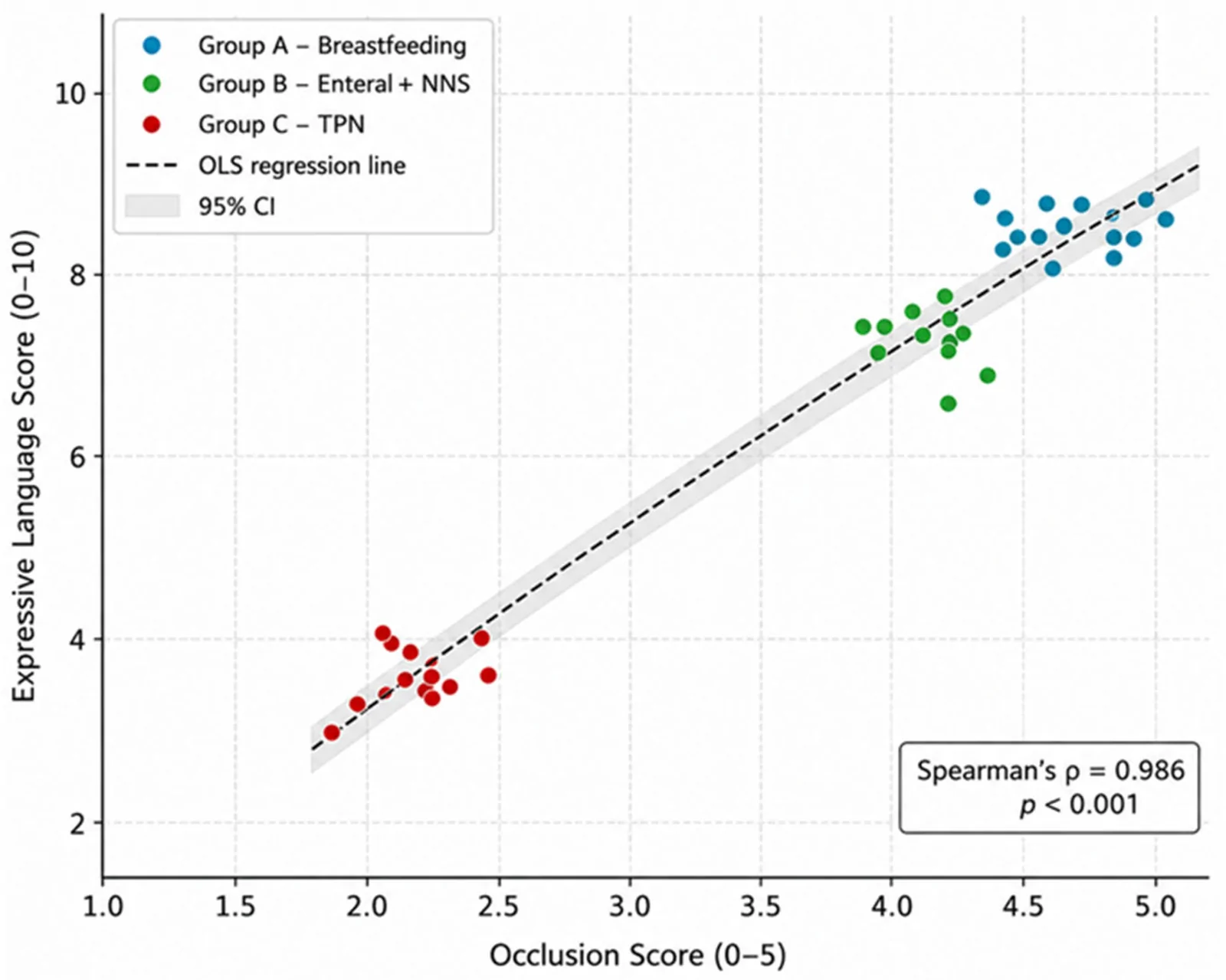
Association between occlusion score and language score according to feeding group. The dashed line represents the overall fitted regression line. (R^2^ ≈ 0.99). Group A = breastfeeding; Group B = enteral feeding with structured non-nutritive sucking; Group C = total parenteral nutrition without structured oral stimulation.

Across all 40 participants, occlusion score and language score were strongly correlated (Spearman ρ = 0.986, *p* < 0.001). Figure 3 shows the scatterplot of individual occlusion and language scores, coloured by feeding group, together with the fitted regression line. In the unadjusted ordinary least squares (OLS) model, each one-point increase in occlusion score was associated with a 2.12-point increase in language score (95% confidence interval [CI] 2.05–2.19), and the model explained 99.0% of the variance in language score (R^2^ = 0.990).

When sex, area of residence and SES were included as covariates, the occlusion coefficient remained essentially unchanged *p* < 0.001), and the adjusted R^2^ remained 0.990 (Table 4). None of the covariates reached statistical significance (sex: *p* = 0.392; residence: *p* = 0.065; SES: *p* = 0.634). Adjustment for sex, area of residence, and socioeconomic status did not materially alter the association between occlusion score and language score. However, residual confounding by unmeasured clinical or environmental factors cannot be excluded. Because the observed overall correlation may partly reflect between-group separation rather than a within-individual relationship, this possibility is considered in the Discussion and represents an important area for future studies.

Regression diagnostics supported the validity of the OLS model. Residuals were approximately normally distributed on Shapiro–Wilk testing and showed no major deviations on quantile–quantile plots; residual-versus-fitted plots did not reveal systematic patterns, and the Breusch–Pagan test did not indicate important heteroscedasticity. Variance inflation factors were low for all covariates, suggesting no problematic multicollinearity, and no influential observations were identified based on Cook’s distance or leverage values. Model stability was explored using leave-one-out cross-validation (LOOCV) and non-parametric bootstrap resampling, which produced regression coefficients and confidence intervals closely matching the primary model. To evaluate whether the strong pooled association might be driven solely by separation between feeding groups, within-group Spearman correlations were also calculated.

Regression diagnostics did not identify major violations of the assumptions of the fitted model. Residuals were approximately normally distributed, and no evidence of substantial multicollinearity, heteroscedasticity, or influential observations was identified (Table 5).

The interaction between sex and occlusion score was not statistically significant (β = 0.04, *p* = 0.58). Sensitivity analyses yielded findings consistent with the primary analyses, supporting the robustness of the observed associations.

### 3.5. Occlusal Morphology Subtypes

**Figure 4:**
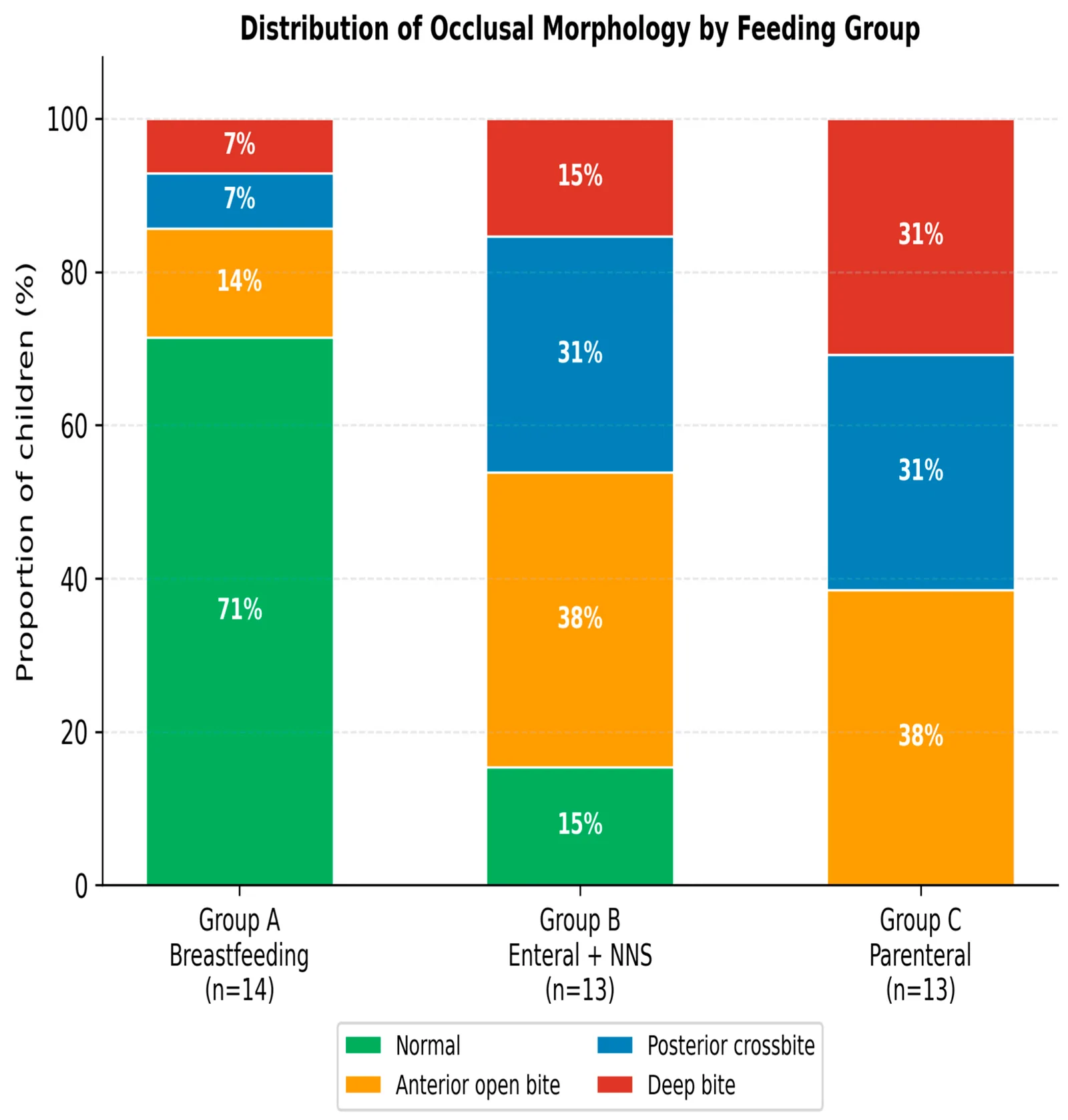
Distribution (%) of occlusal morphology categories according to neonatal feeding modality.

The distribution of occlusal morphology subtypes also differed significantly across the three feeding groups (χ^2^(6) = 19.07, *p* = 0.004, Cramér’s V = 0.49; Figure 4), indicating a moderate-to-large association between neonatal feeding modality and occlusal morphology. Normal occlusion was the predominant finding in Group A (71%), but was observed in only 15% of Group B children and in none of those in Group C. Deep bite, which was absent in the breastfed group, was present in 31% of parenterally fed children. Posterior crossbite showed a similar gradient, occurring in 7% of Group A compared with 31% of Groups B and C.

Figure 4 illustrates these differences.

## 4. Discussion

### 4.1. Key Results

This cross-sectional study of 40 preterm children found that neonatalwith differences in occlusal morphology, expressive language performance, and age at first-word acquisition. Large effect sizes were observed for all three primary outcomes (ε^2^ = 0.81–0.90). Children in the breastfeeding group (Group A) showed the most favorable outcomes, those receiving enteral feeding with structured non-nutritive sucking (Group B) had intermediate outcomes, whereas children receiving total parenteral nutrition without structured oral stimulation (Group C) showed the least favorable outcomes (Spearman’s ρ = 0.986). However, this association should be interpreted and the possibility of residual confounding. Although adjustment for sex, area of residence, and socioeconomic status produced minimal changes in the regression estimates, the influence of unmeasured neonatal, familial, and environmental factors cannot be excluded. Accordingly, the present findings should be interpreted as exploratory associations that generate hypotheses warranting further investigation rather than as evidence of causal relationships between neonatal feeding modality, occlusal develop-ment, and language outcomes.

### 4.2. Mechanical Drive of Craniofacial Development

The functional matrix hypothesis, originally proposed by Moss (1997) [12] and subsequently supported by experimental animal and human studies by Kiliaridis (1995) [13], suggests that craniofacial skeletal growth is influenced primarily by the functional demands imposed by the surrounding soft tissues. Several biological mechanisms have been proposed to explain the associations between early feeding and craniofacial development [12,13,14]. However, these mechanisms were not directly investigated in the present study and are discussed here solely as biological context rather than as conclusions derived from our findings. From a functional perspective, the muscles of mastication and the tongue form an integrated functional matrix. Their activity generates mechanical forces that stimulate sutural growth and periosteal bone apposition, whereas reduced functional loading may attenuate skeletal development. During breastfeeding, coordinated sucking requires repeated activation of the masseter, temporalis, buccinator, and orbicularis oris muscles, while prolonged tube feeding or total parenteral nutrition provides substantially less physiological oral stimulation. Supporting this concept, Liang et al. (2024) [14] used finite-element modelling of infant crania to demonstrate that masticatory forces increase markedly during early childhood and progressively concentrate mechanical strain on the developing facial skeleton. The graded differences in occlusal morphology observed across the three feeding groups in the present study are consistent with previous reports suggesting that breastfeeding supports normal craniofacial development through repeated functional stimulation of the stomatognathic system. Children who received structured non-nutritive sucking during tube feeding showed intermediate outcomes, whereas those receiving total parenteral nutrition exhibited the least favourable occlusal development. Similarly, children in the breastfeeding group had the highest occlusion scores, whereas those in the parenteral nutrition group had the lowest scores. Nevertheless, the present study did not directly assess muscular activity, craniofacial loading, palatal dimensions, or longitudinal skeletal growth. Therefore, the proposed mechanical pathway should be regarded as a biologically plausible explanation rather than a mechanism demonstrated by the present cross-sectional study.

### 4.3. Biochemical Contributions of Breast Milk

In addition to the proposed mechanical pathway, the nutritional composition of breast milk may also contribute to both neural and musculoskeletal development. Koletzko et al. (2020) [3] and Martin et al. (2016) [4] reported that breast milk provides docosahexaenoic acid (DHA), arachidonic acid (AA), zinc, selenium, IGF-1, EGF, and immunoglobulins which may contribute to myelination, cortical synaptogenesis, and craniofacial soft-tissue growth during a highly plastic phase of postnatal development. These biochemical pathways, while biologically plausible and supported by previous work [3,4,5,17,18,19,28,29], were not directly measured in the present cohort and should therefore be interpreted as hypotheses rather than mechanisms demonstrated by our findings.

Nandivada et al. (2021) [5] reported that standard total parenteral nutrition (TPN) formulations may not fully provide the nutritional profile of human milk, while Morris et al. (2022) [31] found that, despite improvements in parenteral nutrition protocols, measurable differences in visual and cognitive outcomes persisted during early childhood. At the micronutrient level, the meta-analysis by Alshaikh et al. (2022) [32] showed that enteral zinc supplementation improved both somatic growth and neurodevelopment in preterm infants, highlighting the potential importance of adequate micronutrient supply during this critical developmental period. Nandivada et al. (2021) [5] reported that standard total parenteral nutrition (TPN) formulations may not fully provide the nutritional profile of human milk, while Morris et al. (2022) [31] found that, despite improvements in parenteral nutrition protocols, measurable differences in visual and cognitive outcomes persisted during early childhood. At the micronutrient level, the meta-analysis by Alshaikh et al. (2022) [32] showed that enteral zinc supplementation improved both somatic growth and neurodevelopment in preterm infants, highlighting the potential importance of adequate micronutrient supply during this critical developmental period. In the present study, children in Group C were exposed to both the absence of physiological oral stimulation and exclusive parenteral nutrition during the neonatal period. Although this combination may represent a biologically plausible explanation for the less favourable occlusal and language outcomes observed in this group, the present study was not designed to determine the relative contribution of nutritional and mechanical factors. Furthermore, Lockyer et al. (2021) [17] reported that maternal micronutrient status influences breast-milk composition and may affect early neurodevelopment. In addition, randomised clinical trials by Gould et al. (2022) [18] and Guillot et al. (2022) [19] reported beneficial effects of DHA supplementation on neurodevelopmental outcomes in preterm infants. However, the present study did not assess nutrient intake, breast-milk composition, circulating micronutrient concentrations, DHA or AA status, or markers of neurological maturation. Consequently, it is not possible to determine whether nutritional factors contributed directly to the observed differences in occlusal morphology or language development. The observed associations may instead reflect the combined influence of feeding modality, neonatal clinical condition, oral-motor stimulation, and other unmeasured developmental or environmental factors.

The present findings are consistent with the concept that early nutrition contributes to the coordinated function of the digestive and stomatognathic systems, helping to maintain physiological homeostasis during a critical period of growth and development. Previous studies have suggested that adequate nutritional and functional oral stimulation supports both craniofacial maturation and neurodevelopment, although these mechanisms were not directly evaluated in the present study [3,12,13,14].

### 4.4. Association Between Occlusion and Language Development

An important association was observed between occlusion and expressive language scores across the study cohort. This association remained largely unchanged after adjustment for sex, area of residence, and socioeconomic status, suggesting that it was not materially influenced by the measured demographic covariates. Nevertheless, the strength of this association should be interpreted with caution. The relatively small sample size, the clear separation between the feeding groups, and the observational cross-sectional design may have contributed to the high correlation coefficient (Spearman’s ρ = 0.986) and the high coefficient of determination observed in the regression model (adjusted R^2^ = 0.990). Consequently, these estimates should be considered cohort-specific and require confirmation in larger prospective multicentre studies.

Expressive language development followed the same gradient observed for occlusal morphology. Children in the breastfeeding group achieved the highest language scores and acquired their first words earlier than those receiving enteral feeding with structured non-nutritive sucking or total parenteral nutrition. This parallel pattern is consistent with the hypothesis that early feeding experiences may influence both orofacial development and early communication skills. Previous neurofunctional models have suggested that orofacial structure and motor control contribute to speech production and articulatory performance [20,21,22]. Although the present findings are compatible with this hypothesis, they do not demonstrate structural mediation or establish a causal relationship between occlusal morphology and language acquisition. Longitudinal studies incorporating mediation analyses are needed to clarify the temporal and functional relationships between these outcomes.

### 4.5. Non-Nutritive Oral Stimulation as a Partial Substitute

This pattern is consistent with previous randomized evidence from Alidad et al. (2021) [23] and the meta-analysis by Zhao et al. (2024) [24], which reported that structured non-nutritive sucking (NNS) and oral-motor stimulation in neonatal intensive care units (NICUs) can improve feeding readiness and facilitate the transition to oral feeding in preterm infants.

The present findings suggest that structured NNS may partially compensate for the reduced oral-motor stimulation associated with tube feeding. However, the present observational study cannot determine whether NNS itself was responsible for the observed differences. Feeding modality was determined by clinical circumstances rather than randomisation, and the groups may have differed in neonatal illness severity and other unmeasured clinical characteristics. Consequently, the intermediate outcomes observed in Group B should be interpreted as an observed group pattern rather than as evidence of a causal effect of NNS.

Although breastfeeding remained associated with the most favourable outcomes, followed by enteral feeding with structured NNS and total parenteral nutrition, these findings suggest that the potential long-term role of NNS in occlusal and language development warrants further investigation in adequately powered prospective studies [25,26,27,28,29,30,31,32].

### 4.6. Relationship to Previous Evidence

The occlusal findings are consistent with previous studies reporting an association between longer breastfeeding duration and a lower prevalence of malocclusion, particularly posterior crossbite [8,9,10,11]. Likewise, previous studies suggest that reduced oral-motor activity may influence craniofacial development through altered functional loading of the stomatognathic system [13,14]. The language findings are also in agreement with epidemiological studies reporting positive associations between breastfeeding and neurodevelopmental outcomes [1,33]. However, language development is multifactorial; therefore, the present study cannot determine whether occlusal development directly influences language acquisition or whether both outcomes reflect common early-life exposures [34,35,36,37,38,39]. Children receiving structured non-nutritive sucking (Group B) consistently showed intermediate outcomes between the breastfeeding and total parenteral nutrition groups. This pattern is consistent with previous studies showing that structured oral stimulation improves feeding readiness and facilitates the transition to oral feeding in preterm infants [23,24,26,27,28]. However, evidence regarding its long-term effects on craniofacial and language development remains limited, and the present findings should therefore be regarded as hypothesis-generating. Finally, regression diagnostics did not identify major violations of model assumptions, supporting the appropriateness of the selected analytical approach. Nevertheless, given the observational design and relatively small sample size, the regression findings should be interpreted cautiously and considered exploratory rather than confirmatory [40,41].

### 4.7. Confounding and Limitations

Several limitations should be acknowledged. First, the study included a relatively small sample of 40 preterm children (13–14 participants per group). Although large effect sizes were observed, estimates derived from small samples should be interpreted cautiously, particularly the notably strong association between occlusal and language scores, which may partly reflect the marked separation between the feeding groups.

Second, feeding modality was determined by clinical necessity rather than random assignment. Consequently, children receiving prolonged enteral or parenteral nutrition were likely to have greater neonatal illness severity, making feeding modality a potential marker of clinical vulnerability rather than an independent developmental exposure [5,6,7]. Because neonatal illness severity scores were unavailable, this potential source of residual confounding could not be assessed directly.

Third, although the regression analysis adjusted for sex, area of residence, and socioeconomic status, other potentially important variables—including gestational age, birth weight, duration of neonatal intensive care, neurological complications, parental education, home language environment, breastfeeding duration, pacifier use, and oral-motor stimulation—were not comprehensively assessed [11,15]. Residual confounding therefore cannot be excluded.

Finally, the cross-sectional design precludes causal inference and cannot determine whether the observed differences persist throughout childhood. Accordingly, the present findings should be interpreted as exploratory associations that require confirmation in larger prospective multicentre studies.

Consequently, the present data should be interpreted as demonstrating associations rather than demonstrating that that feeding modality per se causes the observed differences in occlusion and language outcomes [29,30,31,32,34,35,36].

### 4.8. Generalisability

The findings of this study are primarily applicable to preterm children attending structured developmental follow-up programmes in a Romanian university-based setting. Differences in neonatal care protocols, feeding practices, population characteristics, and access to developmental services may limit the generalisability of these findings to other settings. Although the direction of the observed associations may be relevant to other preterm populations, the magnitude of the reported effects should be interpreted cautiously and should not be assumed to be directly reproducible. Likewise, these findings cannot be extrapolated to term-born children, whose neonatal feeding exposures and baseline risks of occlusal and language disorders differ substantially. Confirmation in larger, multicentre prospective studies involving more diverse populations is therefore warranted.

### 4.9. Clinical Implications

The present findings support considering occlusal morphology and language development as complementary components of developmental follow-up in preterm children. Infants with prolonged absence of oral feeding or early feeding difficulties may benefit from multidisciplinary assessment involving neonatologists, pediatric dentists, and speech-language pathologists.are consistent with previous evidence suggesting a potential role in improving feeding readiness in preterm infants [23,24], although long-term effects on occlusal and language outcomes have not been established. However, the present study was not designed to determine whether non-nutritive sucking prevents later malocclusion or language delay, and these potential long-term effects require confirmation in prospective studies. Accordingly, the present findings are consistent with existing recommendations to promote breastfeeding where clinically feasible and to provide structured oral stimulation when oral feeding is delayed, whilst acknowledging that the present observational data cannot establish the effectiveness of these practices on the specific outcomes studied. Accordingly, the present findings are consistent with existing recommendations promoting breastfeeding whenever possible and structured oral stimulation when oral feeding is delayed.

#### Future Direction

Larger prospective multicenter studies are required to examine occlusal and language development from the neonatal period through school age. Future analyses should include gestational age, birth weight, neonatal illness severity, neurological complications, type and duration of nutritional exposure, parental education, and measures of the home language environment. Studies with repeated assessments could determine whether occlusal and language trajectories develop in parallel and whether their association persists within feeding groups. Mediation analyses may help distinguish whether occlusal morphology contributes independently to language outcomes or whether both primarily reflect common neonatal and environmental factors.

Randomised studies may evaluate the effects of structured oral-motor interventions when ethically and clinically appropriate. External validation in independent cohorts is also required before the present statistical estimates can be considered stable or clinically predictive.

## 5. Conclusions

In this cross-sectional study of 40 preterm children, neonatal feeding modality was associated with differences in occlusal morphology, expressive language performance, and age at first-word acquisition. Breastfed children showed the most favourable outcomes, children receiving enteral feeding with structured non-nutritive sucking (NNS) showed intermediate outcomes, and those receiving total parenteral nutrition without structured oral stimulation had the least favourable outcomes.

Occlusal morphology and expressive language scores were associated across the study cohort, and this issue remained largely unchanged after adjustment for sex, area of residence, and socioeconomic status. However, the strength of this association should be interpreted cautiously in view of the relatively small sample size, the marked separation between feeding groups, and the likelihood of residual confounding from unmeasured neonatal and environmental factors.

The present findings do not establish causal relationships between feeding modality, occlusal development, and language outcomes, nor do they demonstrate a preventive effect of structured non-nutritive sucking. Rather, they support the hypothesis that early feeding experiences may be associated with both craniofacial and communicative development in preterm children.

Larger prospective multicenter studies with comprehensive adjustment for neonatal and environmental confounders are required to confirm these findings and to clarify the long-term relationships between neonatal feeding, occlusal development, and language acquisition.

## Figures and Tables

**Table 1:** Demographic characteristics of the study sample by feeding group (n = 40).

Variable Characteristics	Group A (n = 14)	Group B (n = 13)	Group C (n = 13)	*p*-Value
Sex, M/F	6/8	6/7	7/6	0.844
Residence, Urban/Rural	8/6	8/5	8/5	0.964
SES, High/Medium/Low	6/6/2	2/6/5	0/4/9	0.018
Missing data, n	0	0	0	—

Values are presented as counts, absolute frequencies (n) or raw ratios. SES = socioeconomic status. *p*-values were obtained using the chi-square test.

**Table 2:** Primary outcomes by feeding group—median (IQR) and Kruskal-Wallis test results.

Outcome	Group A (n = 14) Median [IQR]	Group B (n = 13) Median [IQR]	Group C (n = 13) Median [IQR]	Kruskal–Wallis(H)	*p*-Value	ε^2^
Occlusion score (0–5)	4.50 [4.43–4.67]	4.20 [4.10–4.30]	2.30 [2.20–2.40]	H = 33.1;	<0.001	0.81
Language score (0–10)	8.40 [8.22–8.57]	7.20 [7.10–7.50]	3.50 [3.40–3.70]	H = 34.7;	<0.001	0.88
Age at first words (months)	11 [10,11]	15 [15,16]	24 [22–24]	H = 35.3;	<0.001	0.90

Values are presented as median [IQR]. Group differences were assessed using the Kruskal–Wallis test. ε^2^ = epsilon-squared effect size.

**Table 3:** Dunn–Bonferroni post hoc comparisons between feeding groups.

Comparison	Difference in Medians	95% CI (Bonferroni)	*p* (Adjusted)
Occlusion: A vs. B	−0.34	[−0.48, −0.21]	<0.001
Occlusion: A vs. C	−2.21	[−2.34, −2.08]	<0.001
Occlusion: B vs. C	−1.87	[−2.00, −1.74]	<0.001
Language: A vs. B	−1.15	[−1.35, −0.95]	<0.001
Language: A vs. C	−4.86	[−5.06, −4.66]	<0.001
Language: B vs. C	−3.72	[−3.92, −3.51]	<0.001
First words: A vs. B	+4.23	[3.52, 4.93]	<0.001
First words: A vs. C	+13.07	[12.37, 13.77]	<0.001
First words: B vs. C	+8.85	[8.13, 9.56]	<0.001

**Table 4:** Linear regression models predicting language score: unadjusted and adjusted estimates with 95% CI.

Predictor	Model 1 β (95% CI)	Model 2 β (95% CI)	*p*-Value
Occlusion score	2.12 (2.05–2.19)	2.10 (2.01–2.19)	<0.001
Sex	—	−0.06 (−0.19 to 0.08)	0.392
Residence	—	−0.17 (−0.34 to 0.01)	0.065
SES	—	0.03 (−0.09 to 0.14)	0.634

Model 1: R^2^ = 0.990. Model 2 (adjusted for sex, residence and socioeconomic status): adjusted R^2^ = 0.990. CI = confidence interval; SES = socioeconomic status. The high coefficient of determination (R^2^ = 0.990) reflects the strong association observed within this study cohort. Given the relatively small sample size, the marked separation between feeding groups, and the observational study design, these estimates should be interpreted cautiously and regarded as cohort-specific rather than evidence of a causal or universally applicable relationship.

**Table 5:** Diagnostic Tests for the Multivariable Regression Model.

Diagnostic	Assessment	Interpretation
Residual normality	Assessed (Shapiro–Wilk test and Q–Q plot)	No major deviation from normality observed
Homoscedasticity	Residual-versus-fitted plot	No substantial heteroscedasticity observed
Multicollinearity	Variance Inflation Factor (VIF)	No problematic multicollinearity detected
Influential observations	Cook’s distance	No influential observations identified

## Data Availability

The dataset is available from the corresponding author upon reasonable request.

## Supplementary Materials

The following supporting information can be downloaded at: https://www.mdpi.com/article/10.3390/diagnostics16172727/s1, Supplementary File S1: STROBE Checklist for Cross-Sectional Studies; Supplementary File S2: Participant Flow Diagram; Supplementary File S3: Blank Informed Consent Form (Romanian and English Versions).

## Abbreviations

The following abbreviations are used in this manuscript.

AA	Arachidonic acid
CDI	Communicative Development Inventories (MacArthur–Bates)
CI	Confidence interval
DHA	Docosahexaenoic acid
EGF	Epidermal growth factor
GA	Gestational age
IGF-1	Insulin-like growth factor 1
IQR	Interquartile range
LOOCV	Leave-one-out cross-validation
NICU	Neonatal intensive care unit
NNS	Non-nutritive sucking
OLS	Ordinary least squares
OR	Odds ratio
SES	Socioeconomic status
STROBE	Strengthening the Reporting of Observational Studies in Epidemiology
SS	Stomatognathic system
TPN	Total parenteral nutrition
VIF	Variance inflation factor
WHO	World Health Organization
ρ	Spearman’s rank correlation coefficient
