# Supplementary material for: An Exploratory Assessment of Early Feeding Modality and Associated Stomatognathic, Occlusal, and Language Outcomes in Preterm Children: An Observational Cross-Sectional Study
Source: Diagnostics (Basel). 2026 Aug 26;16(17):2727. doi: 10.3390/diagnostics16172727 (PMC13564592; doi:10.3390/diagnostics16172727)
Supplement: Supplementary file 1 [file diagnostics-16-02727-s001.zip › Supplementary File S3 ICS.pdf]

UNIVERSITATEA DE MEDICINĂ ȘI FARMACIE „GRIGORE T. POPA” IAȘI
FACULTATEA DE MEDICINĂ DENTARĂ
DISCIPLINA PEDODONȚIE
în colaborare cu
SPITALUL CLINIC DE URGENȚĂ PENTRU COPII „SF. MARIA” IAȘI

FORMULAR DE CONSIMȚĂMÂNT INFORMAT PENTRU PARTICIPAREA LA UN STUDIU DE CERCETARE

Titlul studiului

Diagnosticul precoce și profilaxia disfuncțiilor sistemului stomatognat: relația dintre modalitatea de alimentație neonatală, dezvoltarea ocluzală și achiziția limbajului la copiii născuți prematur.

Investigator principal

Dr. Lucian Ștefan Burlea/ Dr. Ciubară Ana Maria / Dr . Ana Elena Sîrghie

Instituții participante

- Universitatea de Medicină și Farmacie „Grigore T. Popa” Iași – Facultatea de Medicină Dentară
- Spitalul Clinic de Urgență pentru Copii „Sf. Maria” Iași
- Disciplina Pedodonție – Facultatea de Medicină Dentară

Stimate părinte / reprezentant legal

Sunteți invitat(ă) să permiteți participarea copilului dumneavoastră la un studiu de cercetare. Participarea este voluntară și refuzul sau retragerea din studiu nu va influența actul medical sau îngrijirea copilului.

Scopul studiului

Studiul urmărește evaluarea influenței modalității de alimentație neonatală asupra dezvoltării sistemului stomatognat, dezvoltării ocluzale și achiziției limbajului la copiii născuți prematur.

Proceduri

Participarea presupune analiza istoricului medical, examen clinic stomatologic, evaluarea ocluziei, evaluarea funcțiilor oro-faciale și a dezvoltării limbajului. Nu se efectuează proceduri invazive și nu se administrează tratamente experimentale.

Riscuri

Examinările sunt neinvazive, nedureroase și nu implică riscuri suplimentare față de un consult stomatologic pediatric obișnuit.

Beneficii

Participarea poate permite identificarea precoce a unor tulburări de dezvoltare și formularea unor recomandări preventive sau terapeutice.

Confidențialitatea datelor

Datele vor fi codificate și utilizate exclusiv în scop științific, cu respectarea Regulamentului (UE) 2016/679 (GDPR). Identitatea copilului nu va fi dezvăluită în publicații.

Participare voluntară

Puteți retrage copilul din studiu în orice moment, fără consecințe asupra îngrijirii medicale.

Declarația părintelui / reprezentantului legal

Declar că am citit și am înțeles informațiile de mai sus, am avut posibilitatea de a adresa întrebări, am primit răspunsuri satisfăcătoare și îmi exprim acordul liber și informat pentru participarea copilului meu la acest studiu.

Numele și prenumele copilului: ________________________________

Data nașterii: ____

Numele și prenumele părintelui / reprezentantului legal:

____

Telefon: ____

Adresă e-mail (opțional): ____

☐ DA ☐ NU

Semnătura părintelui / reprezentantului legal: __________________________

Data: ____ / ____ / ______

Investigator: ____

Semnătura investigatorului: _____________________________

Data: ____ / ____ / ______

English variant

"GRIGORE T. POPA" UNIVERSITY OF MEDICINE AND PHARMACY IAȘI
FACULTY OF DENTAL MEDICINEDEPARTMENT OF PEDIATRIC DENTISTRY
in collaboration with
SF. MARIA" CLINICAL EMERGENCY HOSPITAL FOR CHILDREN, IAȘI

INFORMED CONSENT FORM FOR PARTICIPATION IN A RESEARCH STUDY

Study Title

Early Diagnosis and Prophylaxis of Stomatognathic Dysfunction: The Interplay Between Feeding Modality, Occlusal Development, and Language Acquisition in Preterm Children—An Observational Cross-Sectional Study

---

Principal Investigators

- **Dr. Lucian Ștefan Burlea**
- **Dr. Ana Maria Ciubară**
- **Dr. Ana Elena Sîrghe**

Participating Institutions

- "Grigore T. Popa" University of Medicine and Pharmacy Iași – Faculty of Dental Medicine
- "Sf. Maria" Clinical Emergency Hospital for Children, Iași
- Department of Pediatric Dentistry, Faculty of Dental Medicine

---

Dear Parent / Legal Guardian,

You are invited to allow your child to participate in a research study. Participation is entirely voluntary, and refusing to participate or withdrawing your child from the study at any time will not affect the medical care or treatment provided to your child.

Purpose of the Study

The purpose of this study is to evaluate the influence of neonatal feeding modality on the development of the stomatognathic system, occlusal development, and language acquisition in children born preterm.

Study Procedures

Participation in this study includes:

- review of the child's medical and neonatal history;
- clinical dental examination;
- assessment of dental occlusion;
- evaluation of orofacial functions;
- assessment of language development.

No invasive procedures will be performed, and no experimental treatments will be administered.

Risks

The examinations are non-invasive, painless, and do not involve any additional risks beyond those associated with a routine pediatric dental examination.

Benefits

Participation may contribute to the early identification of developmental disorders and facilitate appropriate preventive or therapeutic recommendations whenever necessary.

Confidentiality

All collected information will remain strictly confidential.

Data will be coded and processed exclusively for scientific research purposes in accordance with the **General Data Protection Regulation (EU) 2016/679 (GDPR)**.

The identity of the child will never be disclosed in scientific publications or presentations.

Voluntary Participation

Participation is entirely voluntary.

You may withdraw your child from the study at any time without any consequences for your child's current or future medical care.

Parent / Legal Guardian Declaration

I declare that:

- I have read and understood the information provided above.
- I have had the opportunity to ask questions and have received satisfactory answers.
- I freely and voluntarily agree to my child's participation in this research study.

Child's Full Name

Date of Birth

Parent / Legal Guardian's Full Name

Telephone

E-mail (optional)

☐ **YES**

☐ **NO**

Parent / Legal Guardian Signature

Date

____ / ____ / ______

Investigator

Investigator Signature

Date

____ / ____ / ______
