# Supplementary material for: An Exploratory Assessment of Early Feeding Modality and Associated Stomatognathic, Occlusal, and Language Outcomes in Preterm Children: An Observational Cross-Sectional Study
Source: Diagnostics (Basel). 2026 Aug 26;16(17):2727. doi: 10.3390/diagnostics16172727 (PMC13564592; doi:10.3390/diagnostics16172727)
Supplement: Supplementary file 1 [file diagnostics-16-02727-s001.zip › Supplementary File S2 flow chart.pdf]

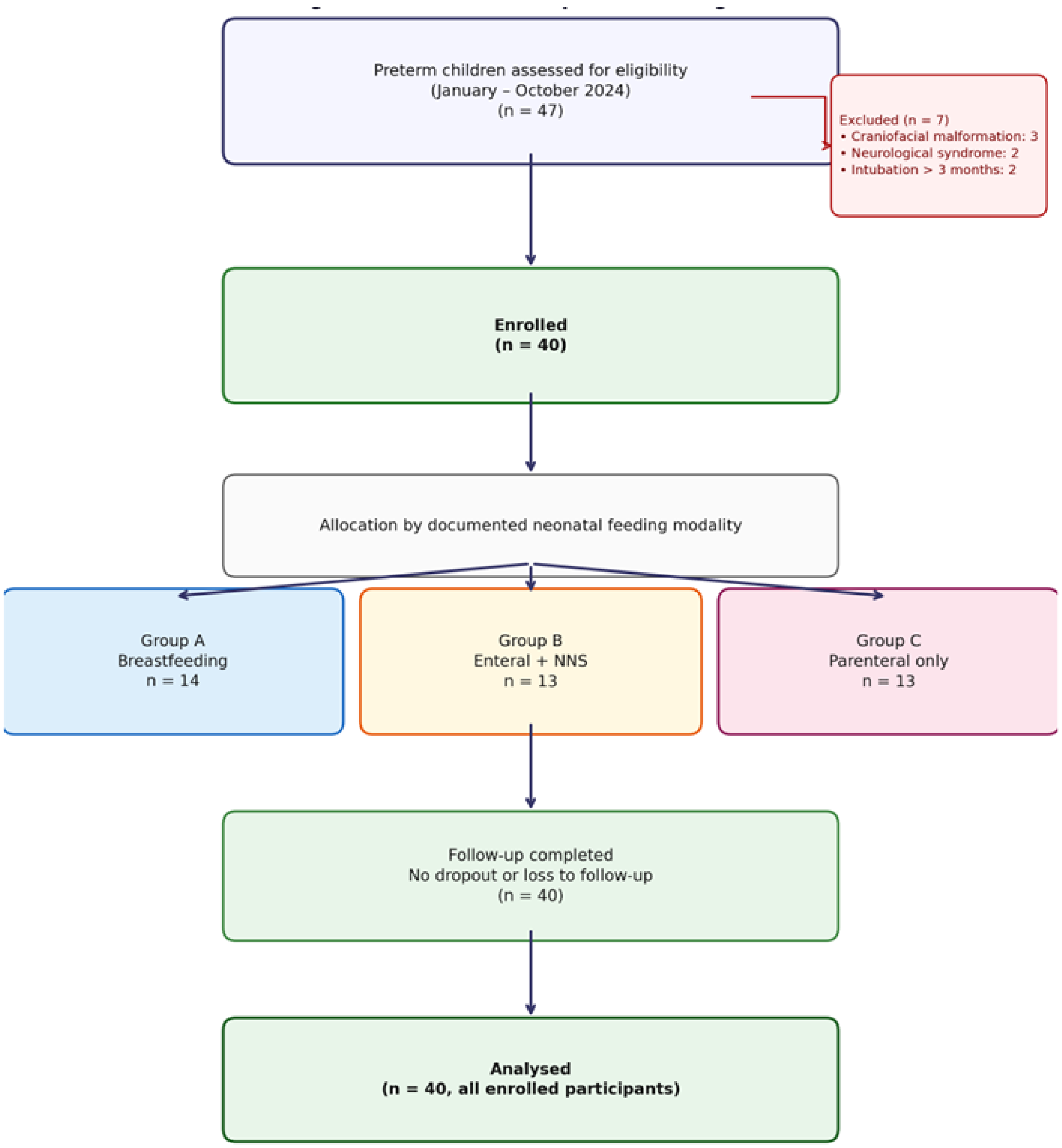
Preterm children assessed for eligibility
(January – October 2024)
(n = 47)
Excluded (n = 7)
• Craniofacial malformation: 3
• Neurological syndrome: 2
• Intubation > 3 months: 2
Enrolled
(n = 40)
Allocation by documented neonatal feeding modality
Group A
Breastfeeding
n = 14
Group B
Enteral + NNS
n = 13
Group C
Parenteral only
n = 13
Follow-up completed
No dropout or loss to follow-up
(n = 40)
Analysed
(n = 40, all enrolled participants)
